# Comparison of endoscopic plantar fasciotomy and endoscopic partial fascia detachment in patients with chronic plantar fasciitis: a retrospective 1-year follow-up study

**DOI:** 10.3389/fsurg.2026.1848295

**Published:** 2026-06-24

**Authors:** Xiao-hui Hu, Jun Li, Ji-yin Tang, Min Li, Rui-jun Bai, Hai-feng Li

**Affiliations:** 1 Department of Orthopaedics, Xuancheng People Hospital, Affiliated Hospital of Wannan Medical College, Xuancheng, Anhui, China; 2 Department of Medical Insurance, Wuxi Ninth People’s Hospital, Soochow University, Wuxi, Jiangsu, China; 3 Department of Orthopaedics, Wuxi Ninth People’s Hospital, Soochow University, Wuxi, Jiangsu, China; 4 Minimally Invasive Orthopedic Institute of Soochow University, Soochow University, Wuxi, Jiangsu, China

**Keywords:** endoscopy, partial plantar fascia detachment, partial plantar fasciotomy, plantar fasciitis, therapeutic effect

## Abstract

**Background:**

Plantar fasciitis (PF) is a common disease that causes pain and dysfunction in the heel. Non-surgical treatments of PF can handle most cases, but recurring pain remains a challenge.

**Purpose:**

This retrospective study evaluated the clinical outcomes of endoscopic plantar fasciotomy and endoscopic partial plantar fascia detachment in patients with PF.

**Study design:**

A single-center retrospective comparative study was conducted on 33 patients with refractory PF between January 2021 and June 2023.

**Methods:**

There were 14 patients in the endoscopic plantar fasciotomy group and 19 patients in the endoscopic partial plantar fascia detachment group. Clinical outcomes were assessed using the visual analog scale (VAS), American Orthopaedic Foot and Ankle Society (AOFAS) score, and 36-item Short-Form Health Survey (SF-36) questionnaire. All patients were evaluated postoperatively and at 3 months, 6 months, and 1 year after surgery.

**Results:**

The two groups showed no differences in age, disease duration, osteophyte, body mass index, and operation time. All patients achieved satisfactory postoperative scores on VAS, AOFAS, and SF-36. Postoperative comparisons between the plantar fasciotomy group and the partial plantar fascia detachment group revealed VAS scores of 4.76 ± 1.49 vs. 2.97 ± 1.30 [mean difference: −1.79, 95% confidence interval (CI): (−6.01, 2.41), *P* = 0.3911]; AOFAS scores of 84.74 ± 1.19 vs. 81.71 ± 1.21 [mean difference: −3.02, 95% CI: (−7.17, 1.12), *P* = 0.0471]; and SF-36 scores of 527.4 ± 10.46 vs. 515.6 ± 11.71 [mean difference: −11.73, 95% CI: (−55.66, 32.20), *P* = 0.0491], respectively. Patients in the endoscopic plantar fasciotomy group had better SF-36 and AOFAS scores at 3 and 6 months after surgery.

**Conclusion:**

Both treatments demonstrated satisfactory clinical outcomes after surgery. However, patients in the endoscopic plantar fasciotomy group achieved earlier recovery and better functional improvement compared with patients in the endoscopic partial plantar fascia detachment group.

## Introduction

Plantar fasciitis (PF) is a degenerative disorder of the plantar fascia and the most common cause of heel pain (1, 2). It predominantly affects people aged 45–65 years, with approximately 10% of the population suffering from this disease (1, 3). PF patients often report severe heel pain that affects walking and reduces quality of life (4–6). In most cases, patients can mitigate and eliminate the symptom through 12 months of conservative treatment (7). Non-operative options include activity modification, ice massage, non-steroidal anti-inflammatory drugs, strengthening exercises, and heel padding (8). However, in 10%–15% of patients, conservative treatments are unsuccessful, necessitating a surgical approach (9, 10). Traditional surgical approaches include plantar fasciotomy, neurotomy, neurolysis, calcaneal spur resection, and calcaneal decompression. At present, however, these treatments are restricted due to their association with skin complications, infections, nerve disorders, persistent pain, prolonged recovery period, and other potential complications (11–13).

Compared with open surgery, endoscopic procedures provide quicker improvement, higher patient satisfaction, and faster return to work. Endoscopic treatment for PF was first reported with good clinical outcomes in 1993 (14). Endoscopic surgery has advantages of less trauma, fewer complications, faster rehabilitation, less postoperative pain, and shorter hospital stay (10, 15). There are a variety of conventional endoscopic approaches. Many scholars have evaluated and reported the effectiveness of endoscopic treatment on PF, but the results vary significantly (14, 16). One study reported that endoscopic plantar fascia release through a modified dual medial deep fascia approach achieved significant clinical improvements in visual analog scale (VAS) scores and American Orthopaedic Foot and Ankle Society (AOFAS) scores in 25 patients with a minimum of 12 months of follow-up (17). Cottom et al. (18) also confirmed that endoscopic debridement of the plantar fascia via a two-portal medial approach was a reliable procedure with effects lasting up to 5 years. While there are plentiful studies on endoscopic surgery for PF with satisfactory clinical outcomes (19–21), there are fewer studies comparing the clinical efficacy of different endoscopic surgical methods. Existing studies comparing endoscopic approaches for PF have primarily focused on distinct technical dimensions. Çatal et al. (22) compared deep fascial and superficial fascial approaches for endoscopic plantar fasciotomy (EPF), while Tang et al. (10) and Dong et al. (23) evaluated the efficacy of different portal entry techniques for endoscopic plantar fascia release or spur resection combined with fasciotomy. However, studies directly comparing the clinical efficacy of standard EPF and endoscopic partial plantar fascia detachment (EPFD) using standardized terminology remain rare. PF can also be induced by other factors, such as overuse stress and heel spurs, with the latter often observed on lateral calcaneus radiographs (24). Arunakul et al. (25) reported patients underwent endoscopic plantar fascia release combined with plantar heel spur resection could achieve improved of postoperative the 36-item Short-Form Health Survey questionnaire SF-36 questionnaires (SF-36), VAS scores and lower incidence of postoperative complications. To our knowledge, the present study is among the first to directly compare the efficacy of EPF and EPFD for refractory PF. Therefore, we attempted to explore the impact of different endoscopic surgical methods on the prognosis of PF. The aim of this study was to evaluate the short-term outcomes of endoscopic plantar fasciotomy and endoscopic partial plantar fascia detachment, performed in conjunction with heel spur removal. In addition, we hypothesized that endoscopic partial plantar fascia detachment treatment would yield functional outcomes and pain levels comparable to endoscopic plantar fasciotomy.

## Patients and methods

### Patients

This retrospective comparative study analyzed patients with refractory PF admitted to Xuancheng People’s Hospital, Affiliated Hospital of Wannan Medical College, between January 2021 and June 2023. The patients were assigned to either the endoscopic plantar fasciotomy group or the endoscopic partial plantar fascia detachment group. All patients has experienced symptoms for at least 6 months and showed poor response to non-operative treatment. The diagnostic criteria for PF were based on prior studies (20). All patients had an approximately 6-month history of heel pain, with no symptom alleviation after oral medication and non-surgical treatment, including anti-inflammatory drugs and physical therapy. Patients with comorbid conditions such as ankle osteoarthritis and rheumatoid arthritis were excluded from the study, as were those with a surgery history of plantar fasciitis surgery, uncured infection in the treatment areas, or corticosteroid injection history in the past 1month.

Prior to surgery, patients were educated about the benefits and drawbacks of the two surgical methods, including incision type, cost, and reported outcomes. Each patient was given the option to choose either endoscopic plantar fasciotomy or endoscopic fascia detachment. For patients who had difficulty deciding, suggestions were provided by the surgeon emphasizing lower cost, minimally invasive technique, and stable outcomes. Then, surgical treatment was performed according to the patient's choice. Of the 33 recruited patients, 14 underwent endoscopic plantar fasciotomy treatment and 19 underwent endoscopic partial plantar fascia detachment treatment.

This study was conducted in accordance with the Declaration of Helsinki and was approved by the ethics committee and institutional review board of the hospital. Informed consent was obtained from all patients.

### Surgical procedures

**Figure 1:**
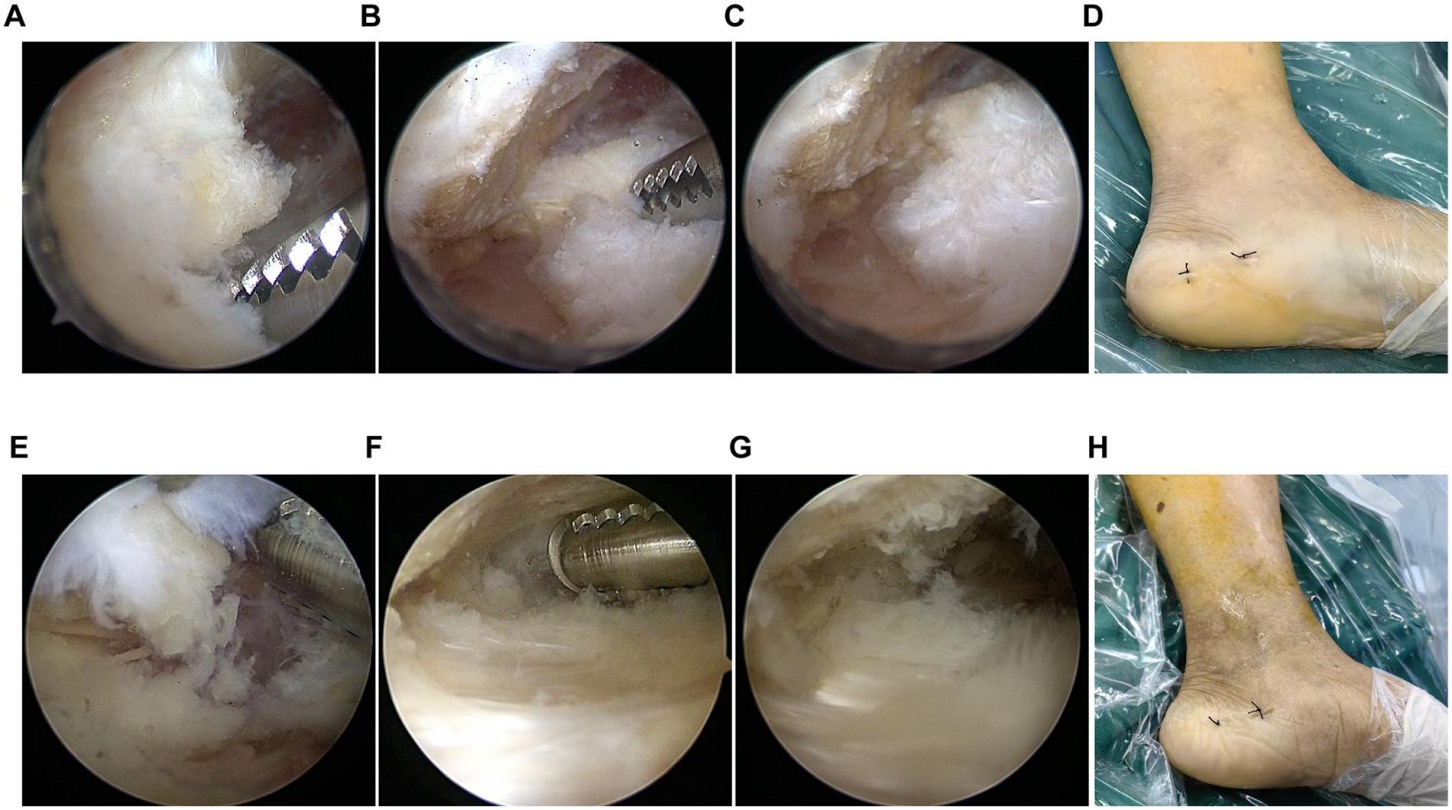
Endoscopic views of plantar fasciotomy and partial plantar fascia detachment. Plantar fasciotomy group: **(A)** Intraoperative endoscopic view of the plantar fascia before excision, showing the intact fascia structure in the plantar fasciotomy group. **(B)** A scabbard grinding drill was used to remove the plantar fascia under the guidance of the positioned needle. **(C)** Intraoperative endoscopic view after the release, visualizing the underlying flexor digitorum brevis muscle and confirming the completion of the fasciotomy. **(D)** Clinical photograph showing the postoperative appearance of the two minimal incisions (portals) with sutures in place, demonstrating the minimally invasive nature of the procedure. Plantar fascia detachment group: **(E)** Intraoperative endoscopic view of the plantar fascia before detachment in the plantar fascia detachment group. **(F)** A scabbard grinding drill was used to release the plantar fascia. **(G)** Intraoperative endoscopic view after the release, visualizing the underlying flexor digitorum brevis muscle and confirming the detachment of fascia. **(H)** Clinical photograph showing the postoperative appearance of the two minimal incisions with sutures in place.

All patients were treated using the Arthrex arthroscope system (Arthrex®, Florida, USA) with a 4.0-mm diameter and 30°angle for minimally invasive surgery. Under general anesthesia, patients were placed in a supine position on the operating table. Two incisions were made on the medial side of the heel. The posterior incision was placed at the intersection of the ventral boundary of the dorsum of the foot and the line tangent to the posterior edge of the medial malleolus, while the anterior incision was positioned 2.0 cm anterior to the posterior incision along the dorsal–ventral border of the foot. After blunt dissection with a trocar, an arthroscope was inserted into the posterior incision to observe the fascia, while a motorized shaver was inserted into the anterior incision. Visualization and manipulation of the plantar fascia were achieved via a dual-portal endoscopic technique, utilizing alternating viewing and working portals to accommodate the endoscope and motorized shaver. In the endoscopic plantar fasciotomy group, inflammatory tissue surrounding the plantar fascia was removed, and the medial one-third of the fascia was excised. An example of a typical endoscopic plantar fasciotomy is shown in Figure 1.

In the endoscopic partial plantar fascia detachment group, incision placement and operative channel establishment were consistent with the fasciotomy group. Debridement focused on inflamed bursae, primarily the loosened inflamed bursae located at the insertion point of the calcaneus. Generally speaking, the loosened inflamed bursae account for 30%–50% of plantar fascia, and the maximum degree of loosening fascia should no further than the midpoint of medial plantar fascia. Images before and after the surgery, as well as of the endoscopic plantar detachment procedure, are shown in Figures 1E–H. In addition, heel spurs if present were removed at the same time for patients of both groups. The removal of heel spurs was achieved via a powered drill under endoscopic guidance, ensuring smoothness consistent with the surrounding cortical bone.

### Postoperative treatment and rehabilitation

All patients followed the same postoperative functional training and rehabilitation program with the assistance of physical therapists. The patients were required to take oral antibiotics and non-steroidal drugs for 3 days to relieve pain and prevent wound infection. Non-weight-bearing functional exercises and lower-limb isometric muscle contraction exercises could be performed on the second day after surgery. Meanwhile, postoperative strength exercises for the muscles around the foot and ankle were also routinely performed under the guidance of rehabilitators. Patients gradually resumed weight-bearing functional exercises and physical activity 1 week after surgery.

### Evaluation

Patient data including age, gender, duration of symptoms, clinical features, family history, follow-up duration, recurrence, and complications were recorded. During the first 3 months after surgery, all patients were interviewed by phone or in person about their weekly recovery state and activity levels. The VAS, AOFAS and the 36-item Short-Form Health Survey questionnaire (26) were evaluated for all patients to assess their prognosis and therapeutic outcomes. All patients completed 100% of the items in each questionnaire at every assessment time point, with no missing responses or incomplete submissions. The main criteria for VAS score are as follows: 7–10 points indicate severe pain; 4–6 points indicate moderate pain; 1–3 points indicate mild pain; and 0 points indicates no pain (27). Postoperative evaluation was performed on the second day after surgery. The time points of postoperative follow-up were at 3 months, 6 months, and 1 year. Furthermore, the incision was evaluated by the surgeon immediately after the wound had healed. All postoperative measurements were conducted by the same single rehabilitation physician, who was not involved in the surgery and was double-blinded to both the patient group allocation and the specific surgical procedure performed. Notably, interobserver reliability testing was not performed in this study, as all assessments were completed by this single double-blinded evaluator.

### Statistical analysis

Data were analyzed using SPSS 19.0 (IBM®, New York, NY, USA) statistical software. Quantitative variables were expressed as mean ± standard deviation. The Shapiro–Wilk test was used to assess whether the variables follow a normal distribution. For between-group comparisons, Student’s *t*-test was used for normal distribution and the Mann–Whitney test was used for asymmetric distribution. For all between-group comparisons of continuous variables, mean differences (95% confidence interval, CI) were calculated and reported alongside *P*-values to quantify the magnitude and precision of treatment effects. The Pearson *χ*^2^ test compared the categorical variables. A *P*-value less than 0.05 was considered statistically significant.

## Results

A total of 33 patients were treated at Xuancheng People’s Hospital, Affiliated Hospital of Wannan Medical College, between January 2021 and June 2023. A total of 14 patients underwent endoscopic plantar fasciotomy and 19 patients underwent endoscopic partial plantar fascia detachment. Basic demographic and disease characteristics of these two groups are presented in Table 1. These two groups showed no differences in age, disease duration, osteophyte presence, body mass index (BMI), operation time, or other accompanying diseases, as shown in Tables 1, 2. We also conducted the subgroup analysis and found that age, disease duration, and BMI did not affect the clinical outcome in either group, as shown in Supplementary Table S1.

**Table 1:** Characteristics of the study population.

Variable	Plantar fasciotomy (*n* = 14)	Plantar fascia detachment (*n* = 19)	*P-*value
Age (year ± SD)	57.86 ± 1.45	56.68 ± 2.04	0.6653
Sex (no.)			0.4377
Male	3	10	
Female	11	9	
Side			0.8600
Left	8	8	
Right	6	11	
Disease duration (month ± SD)	6.36 ± 0.31	7.47 ± 0.48	0.0753
Osteophyte (no/%)	6/42.86	12/63.16	
BMI	23.72 ± 0.64	22.30 ± 0.42	0.0635
Operation time (min ± SD)	63.21 ± 3.13	62.11 ± 3.27	0.8138
Follow time (month ± SD)	14.43 ± 2.36	18.22 ± 2.58	0.2980

BMI, body mass index.

**Table 2:** Other diseases of the study population.

Variable	Plantar fasciotomy (*n* = 14)	Plantar fascia detachment (*n* = 19)
Hypertension	2	3
Diabetes	0	1
Coronary heart disease	2	2
Cerebral lacunar infarction	0	1
Anemia	0	1
Pulmonary disease	2	0
Knee osteoarthritis	2	3
Hyperuricemia	1	0
Hepatobiliary lithiasis	2	0
Thyroid disease	1	2

All 33 patients completed the full follow-up, with no cases lost. Notably, complete follow-up was accompanied by 100% completeness of all questionnaire data, with no missing items or incomplete submissions for VAS, AOFAS, or SF-36 assessments across all patients at any evaluation time point. Both groups of patients received good prognoses without incision infection or early complications, such as vascular injury or nerve injury, at the first follow-up. Improvement across all the functional scores, namely, VAS, AOFAS, and SF-36, was recorded preoperatively, postoperatively, and at 3 months, 6 months, and 1 year after surgery. At the final follow-up, all patients reported satisfactory outcomes, with pain reduction or complete pain relief, good walking ability, and normal physical function. We did not observe any other complications, such as worsening pain, long-term collapse of the foot/arch, and lateral foot pain in these two groups of patients.

Postoperative VAS, AOFAS, and SF-36 scores of the endoscopic plantar fasciotomy group were 4.76 ± 1.49, 84.74 ± 1.19, and 527.4 ± 10.46, respectively, while the endoscopic partial plantar fascia detachment group recorded values of 2.97 ± 1.30, 81.71 ± 1.21, and 515.6 ± 11.71, respectively. Furthermore, there were significant differences in AOFAS and SF-36 scores between groups, as shown in Table 3 [mean difference (95% CI): −3.02 (−7.17, 1.12), *P* = 0.0471; and −11.73 (−55.66, 32.20), *P* = 0.0491, respectively]. Preoperative scores showed no differences between groups for VAS, AOFAS, and SF-36. In addition, we also observed significant differences within these two groups when comparing preoperative and postoperative VAS, AOFAS, and SF-36 scores, as shown in Table 3 (*P* ˂0.0001 and *P* = 0.0099, respectively).

**Table 3:** Comparison of the two groups.

Variable	Plantar fasciotomy (*n* = 14)	Plantar fascia detachment (*n* = 19)	Mean difference (95% CI)	*P-*value
VAS				
Preoperative	66.90 ± 1.94	70.25 ± 1.68	3.34 [−1.90, 8.57]	0.2030
Postoperative	4.76 ± 1.49	2.97 ± 1.30	−1.79 [−6.01, 2.41]	0.3911
*P-*value	<0.0001	<0.0001		
AOFAS				
Preoperative	33.05 ± 1.74	35.21 ± 1.71	2.16 [−2.94, 7.26]	0.3941
Postoperative	84.74 ± 1.19	81.71 ± 1.21	−3.02 [−7.17,1.12]	0.0471
*P-*value	<0.0001	<0.0001		
SF−36				
Preoperative	386.7 ± 43.07	423.1 ± 37.81	36.33 [−12.42, 85.09]	0.1387
Postoperative	527.4 ± 10.46	515.6 ± 11.71	−11.73 [−55.66, 32.20]	0.0491
*P-*value	0.0099	<0.0001		

VAS, visual analog scale; AOFAS, American Orthopaedic Foot and Ankle Society; SF-36; the 36-item Short-Form Health Survey questionnaire.

We also evaluated pain scores and functional recovery at 3 months, 6 months, and 1 year after surgery. We found that both groups achieved satisfactory reductions in VAS scores at the 3-month, 6-month, and 1-year follow-up, with no significant differences between groups, as shown in Table 4 [3-month VAS: −1.25 (−4.51, 2.01), *P* = 0.4391; 6-month VAS: −0.91 (−3.73, 1.91), *P* = 0.5157; 1-year VAS: −0.63 (−3.22, 1.97), *P* = 0.6259]. Both groups showed improved SF-36 scores at 3 months, 6 months, and 1 year postoperatively, but the endoscopic plantar fasciotomy group had significantly better scores at the 3-month postoperative evaluation [mean difference: −30.3, 95% CI: (−53.74, −6.86), *P* = 0.0417]. AOFAS score showed improvement for both groups, but the endoscopic fasciotomy group showed superior outcomes at 3 months, 6 months, and 1 year after surgery, as shown in Table 4 [3-month AOFAS: −6.68 (−10.51 to −2.84), *P* = 0.0012; 6-month AOFAS: −6.28, (−10.12, −2.43), *P* = 0.0023; 1-year AOFAS: −4.28 (−8.19, −0.36), *P* = 0.0332].

**Table 4:** Comparison of pain and function evaluation between the two groups.

Variable	Plantar fasciotomy (*n* = 14)	Plantar fascia detachment (*n* = 19)	Mean difference (95% CI)	*P-*value
VAS (mm)				
3 months	3.64 ± 1.31	2.39 ± 1.05	−1.25 [−4.51,2.01]	0.4391
6 months	3.08 ± 0.99	2.17 ± 0.96	−0.91 [−3.73,1.91]	0.5157
1 year	2.74 ± 0.85	2.12 ± 0.93	−0.63 [−3.22, 1.97]	0.6259
AOFAS				
3 months	90.11 ± 1.35	83.43 ± 1.20	−6.68 [−10.51, −2.84]	0.0012
6 months	93.42 ± 1.43	87.14 ± 1.03	−6.28 [−10.12, −2.43]	0.0023
1 year	95.42 ± 1.46	91.14 ± 1.19	−4.28 [−8.19, −0.36]	0.0332
SF−36				
3 months	562.5 ± 15.58	532.2 ± 13.35	−30.3 [−53.74, −6.86]	0.0417
6 months	560.8.0 ± 10.06	556.1 ± 9.38	−4.70 [−42.01, 32.61]	0.2990
1 year	580.6 ± 11.02	566.6 ± 10.02	−13.94 [−45.48, 17.61]	0.3746

VAS, visual analog scale; AOFAS, American Orthopaedic Foot and Ankle Society; SF-36; the 36-item Short-Form Health Survey questionnaire.

## Discussion

Conservative treatment is typically effective for PF patients. However, surgical intervention may be recommended for patients with refractory PF who fail to respond to conservative treatment. In recent years, it has been proved that endoscopic techniques are effective and backed by evidence in the treatment of PF, resulting in satisfactory therapeutic outcomes (22, 28). A large number of scholars have reported that endoscopic surgery has the advantages of good surgical outcomes, low trauma, low recurrence rate, and rapid postoperative recovery (2, 29, 30). Johannsen et al. (31) conducted a randomized controlled trial to explore the effect of operative treatment and non-surgical interventions for PF. They reported significantly superior results for the operative treatment of plantar fasciitis as measured by foot function at 1-year and by VAS activity at 2-year follow-up. In this study, more surgery patients returned to running and jumping activities earlier than the non-operative patients (31). In the present study, we retrospectively analyzed the therapeutic effect of endoscopic treatment for PF patients. We found that endoscopic intervention can achieve good outcomes in both the endoscopic plantar fasciotomy group and the endoscopic partial plantar fascia detachment group. All patients reported significantly improved VAS, AOFAS, and SF-36 functional scores after surgery. These findings align with previous investigations, confirming that surgical intervention is an effective treatment option with successful results for PF. In addition, patients reported comparable functional and scores of VAS, AOFAS, and SF-36 at 6 months and 1 year, suggesting that both techniques can achieve comparable medium-term functional outcomes. Prior studies have reported that risk factors for developing plantar fasciitis include limited ankle dorsiflexion and higher BMI. Researchers also found that runners are more likely to develop this condition because the plantar fascia stretches and contracts while running (8, 32). Jiang et al. (33) studied a four-step method of treatment for plantar fasciitis and found both plantar fasciotomy and partial plantar fascia detachment combined with bone spur removal achieved appropriate and effective clinical outcomes. These studies demonstrated that both plantar fasciotomy and partial plantar fascia detachment are effective treatments for PF. However, most investigations have compared operative treatment versus conservative management or endoscopic surgery versus open techniques. Thus, the present study innovatively builds on previous studies by comparing different endoscopic treatment processes, aiming to identify a more suitable surgical treatment program for PF. In the present study, we found both treatments achieved good clinical outcomes. In addition, endoscopic plantar fasciotomy treatment showed better functional outcomes and an improved pain score at the 3-month follow-up. Meanwhile, endoscopic plantar fasciotomy-treated patients also showed better AOFAS scores than patients in the endoscopic partial plantar fascia detachment group at the 6-month and 1-year postoperative follow-up. This suggests earlier functional recovery in this group. We postulate that the earlier functional recovery in the endoscopic plantar fasciotomy group may be associated with reduced local inflammatory response, while the early pain symptoms in the endoscopic partial plantar fascia detachment group may stem from inflammation and tissue edema. These changes diminished over time, after which the patients reported similar long-term outcomes.

There has been much controversy about whether or not to remove calcaneal spurs during the surgical treatment of PF. Previous studies have stated that heel spurs are not the cause of plantar pain, though most PF patients present with heel spurs in radiographic imaging (34). Davies et al. (34) reported that surgical intervention including partial plantar fascia detachment combined with remove of heel spurs could reduce pain symptom in 76% of patients. However, some scholars still insist that there is no association between heel pain and bone spurs, claiming that removal of the bone spurs along may not alleviate heel pain (35). Kuyucu et al. (36) studied the clinical effects and prognostic factors of PF, noting that calcaneal spurs, age, BMI, symptom duration, and pain sensation are negatively associated with therapeutic outcomes. Previous studies have reported that chronic heel pain associated with bone spurs may be influenced by multiple factors, including spur length (i.e., longer spurs may be more symptomatic) and concurrent fat pad abnormalities (3). Nakajima (37) further confirmed that heel spurs can be pathogenic, with endoscopic calcaneal spur resection resulting in good outcomes, early return to weight-bearing, and few complications in chronic heel pain patients. In the present study, we observed that bone spurs were consistently associated with irritation of the plantar fascia and correlated with heel pain. Therefore, we recommend removal of bone spurs during endoscopic procedures, as spur excision can be performed with minimal trauma and may be conducive to faster recovery.

Our cohort study suggests that the majority of adult patients with similarly defined refractory PF can achieve favorable clinical outcomes through endoscopic intervention. Both endoscopic procedures are minimally invasive, and the dual medial portal approach combined with standardized debridement and spur resection protocols is widely adopted in orthopedic centers, further supporting the transferability of our conclusions to institutions with equivalent technical capabilities.

## Limitations

This study has several limitations. First, this is a single-center retrospective analysis and each group had a relatively small sample size, which may introduce selection bias. Importantly, as a retrospective observational study without randomization, our design precludes the establishment of causal relationships. All reported links between surgical approach and clinical outcomes should be interpreted as associations rather than causal effects. Second, we only compared endoscopic surgical treatments for PF, and other therapies such as open surgical treatment were not included in our study. Third, group assignment was non-randomized and based on patient choice and preference, which may introduce selection bias. Although we standardized preoperative education and confirmed baseline comparability, unmeasured confounders (e.g., patient expectations or pain tolerance) could still influence outcomes. Future randomized controlled trials are needed to eliminate these biases and validate our findings. Fourth, all functional outcome measurements were assessed using subjective scales and completed by a single rehabilitation physician who was double-blinded to both group allocation and surgical procedure. While this double-blinding minimized assessment bias, the lack of multiple independent assessors means interobserver reliability could not be evaluated, which may limit the reliability of the measurement results. Finally, the follow-up period of this study was relatively short, due to which some possible long-term complications may go undetected, such as instability, compensatory tendonitis, or collapse of the foot/arch. Therefore, the long-term therapeutic effects remain to be determined.

## Conclusion

This study demonstrated good outcomes for the treatment of plantar fasciitis using both endoscopic plantar fasciotomy and endoscopic partial plantar fascia detachment combined with heel spur removal. The endoscopic plantar fasciotomy achieved an earlier recovery state and better functional evaluation at the short-term follow-up. However, both treatments achieved appreciable and satisfactory clinical outcomes at the 1-year postoperative follow-up.

## Data Availability

The original contributions presented in this study are included in the article/Supplementary Material, further inquiries can be directed to the corresponding author.
