# Supplementary material for: Comparison of endoscopic plantar fasciotomy and endoscopic partial fascia detachment in patients with chronic plantar fasciitis: a retrospective 1-year follow-up study
Source: Front Surg. 2026 Jun 24;13:1848295. doi: 10.3389/fsurg.2026.1848295 (PMC13342273; doi:10.3389/fsurg.2026.1848295)
Supplement: Supplementary file 1 [file Table1.docx]

| Table-1 The subgroup analysis of study population | | | | |
| --- | --- | --- | --- | --- |
| **Variable** | | **Plantar fasciectomy (n =14)** | **Plantar fascia detachment (n = 19)** | ***P* value** |
| **Age**  **(year**± SD) | ≥ 60 years | 67.86±2.13 | 60.60±3.00 | 0.9518 |
|  | ˂ 60 years | 57.86±1.28 | 52.33±2.23 | 0.2820 |
| **Disease Duration (month**± SD) | ≥ 12 months | 12.43±0.20 | 13.00±0.37 | 0.2355 |
|  | ˂ 12 months | 6.14±0.44 | 6.67±0.40 | 0.4088 |
| **BMI** | ≥ 30 | 33.38±0.75 | 31.85±0.29 | 0.0953 |
|  | ˂ 30 | 23.75±1.18 | 22.45±0.60 | 0.2909 |
| BMI, body mass index; SD, standard deviation. | | | | |
